# Antifungal Activity and Molecular Mechanisms of Copper Nanoforms against *Colletotrichum gloeosporioides*

**DOI:** 10.3390/nano13232990

**Published:** 2023-11-22

**Authors:** Mun’delanji C. Vestergaard, Yuki Nishida, Lihn T. T. Tran, Neha Sharma, Xiaoxiao Zhang, Masayuki Nakamura, Auriane F. Oussou-Azo, Tomoki Nakama

**Affiliations:** 1 Faculty of Agriculture, Kagoshima University, Kagoshima City 890-0065, Japan; 2 Faculty of Fiber Science and Engineering, Kyoto Institute of Technology, Matsugasaki, Sakyo, Kyoto 606-8585, Japan; 3 United Graduate School of Agricultural Sciences, Kagoshima University, Kagoshima City 890-0065, Japan

**Keywords:** copper nanoforms (Cu NFs), antimicrobial, *Colletotrichum gloeosporioides*, giant unilamellar vesicles (GUVs), melanin production

## Abstract

In this work, we have synthesized copper nanoforms (Cu NFs) using ascorbic acid as a reducing agent and polyvinylpyrrolidone as a stabilizer. Elemental characterization using EDS has shown the nanostructure to be of high purity and compare well with commercially sourced nanoforms. SEM images of both Cu NFs show some agglomeration. The in-house NFs had a better even distribution and size of the nanostructures. The XRD peaks represented a face-centered cubic structure of Cu_2_O. The commercially sourced Cu NFs were found to be a mixture of Cu and Cu_2_O. Both forms had a crystalline structure. Using these two types of Cu NFs, an antimicrobial study against *Colletotrichum gloeosporioides,* a devastating plant pathogen, showed the in-house Cu NFs to be most effective at inhibiting growth of the pathogen. Interestingly, at low concentrations, both Cu NFs increased fungal growth, although the mycelia appeared thin and less dense than in the control. SEM macrographs showed that the in-house Cu NFs inhibited the fungus by flattening the mycelia and busting some of them. In contrast, the mycelia were short and appeared clustered when exposed to commercial Cu NFs. The difference in effect was related to the size and/or oxidation state of the Cu NFs. Furthermore, the fungus produced a defense mechanism in response to the NFs. The fungus produced melanin, with the degree of melanization directly corresponding to the concentration of the Cu NFs. Localization of aggregated Cu NFs could be clearly observed outside of the model membranes. The large agglomerates may only contribute indirectly by a hit-and-bounce-off effect, while small structures may adhere to the membrane surface and/or internalize. Spatio-temporal membrane dynamics were captured in real time. The dominant dynamics culminated into large fluctuations. Some of the large fluctuations resulted in vesicular transformation. The major transformation was exo-bud/exo-cytosis, which may be a way to excrete the foreign object (Cu NFs).

## 1. Introduction

Metals have been exploited for their antimicrobial properties for thousands of years. Since the era of Persian kings, copper and silver have been used for food preservation and water sanitization. Copper compounds have been used in agriculture as a fungistatic agent on potatoes and grapes [1]. Nanoforms of metal ions have been reported to increase their antimicrobial properties [2]. These nanoforms possess significant cytotoxicity activity against viruses, fungi, and bacteria. Metal-based nanoparticles (NPs) have the potential to be effective as antimicrobial agents through different mechanisms with respect to the classical treatments and have the potential to be able to target multiple microbes and biomolecules compromising the development of resistant strains [3]. For example, silver, gold, zinc oxide, copper, and copper oxide nanoparticles are commonly used in antibiotherapy. Abbaszadegan et al. demonstrated that positively charged Ag NPs are strongly attracted to the surface of bacteria, resulting in significant antibacterial activity [4]. ZnO NPs and Au NPs possess antimicrobial activity against different Gram-positive (*S. aureus* and many others) and Gram-negative (namely *P. aeruginosa*, *E. coli*, etc.) bacteria [5].

In the synthesis of metal NPs, natural plant extracts can be used both as reductants and stabilizers. They are easily accessible, biocompatible, and eco-friendly, which may synergistically increase the antimicrobial performances of Cu NPs [6]. Furthermore, Cu NPs dissolve faster than other noble metals by releasing ions into the surroundings [7]. Thus, they have found numerous applications in several areas, such as additives in metal coatings, inks, skin products, and plastics for food packaging [8]. Concerning their antimicrobial activity, Cu NPs generate oxidative stress, cause the disassembly of viruses or bacterial membranes, and can interfere with virus activity [9]. At the same time, Cu ions increase the generation of compounds that are toxic for microbes. Copper oxide nanoparticles (CuO NPs) stand out by possessing very well-known antibacterial properties, being active against a varied range of pathogenic bacteria [10]. The integration of metal oxide NPs improves its antimicrobial as well as physical, chemical, and mechanical properties [11]. Cu_2_O NPs showed strong antibacterial properties and effectively damaged the bacterial cell membranes [12].

Metal ions can create strong coordination bonds with atoms (N, O, or S) that are abundantly present in biomolecules and organic compounds. Generally, the bond between metal ions and biomolecules is non-specific, which exhibits a broad-spectrum activity to the metal-based NPs [13].

Multiple studies have investigated the different actions that copper nanoforms (Cu NFs), that is, nanostructured copper (Cu NPs) and its oxidized forms (CuO NPs, Cu_2_O NPs) exert on microbes [14,15]. In general, Cu NPs are more prone to interact with the bacterial membrane, compromising its integrity [14]. Meanwhile, CuO NPs, depending on the shape of the NP, tend to penetrate the bacterial membrane and release ions within the cell [15]. The basic process that takes place for Cu NPs is based on the redox equation, which involves the energetically favorable oxidation of copper to Cu(II) (Cu(s) + O_2_ + 2H^+^ = Cu^2+^ + H_2_O_2_). Thus, the local production of hydrogen peroxide (H_2_O_2_) causes membrane damage [16]. This was in accordance with the cellular membrane study, where exposure to Cu ions caused a minor impact [17]. Another research has suggested that CuO NPs can also produce reactive oxygen species (ROS). The authors showed that *E. coli* is more affected by CuO NPs than *S. aureus*, suggesting that the membrane differences between Gram-negative and Gram-positive bacteria can influence the resistance to ROS [18]. The authors also evidenced a correlation between the amount of killed bacteria and the size of the NP and highlighted that smaller NPs are more effective against bacteria. Furthermore, smaller NPs are associated with an increased amount of superoxide anions, generating more intense oxidative stress. Another study demonstrated that the shape of the NP significantly influences the overall antibacterial activity by comparing the minimum bactericidal concentration and minimum inhibitory concentration of nanosheets of CuO and spherical CuO NPs against *Proteus vulgaris*, *E. coli, Bacillus subtilis*, and *Micrococcus luteous* [19]. When tested in mice, Cu nanocrystals were reported to catalyze the production of the hydroxyl radical to kill the bacteria and hinder the further production of biofilms [20]. Cu NPs and CuO NPs have shown antimicrobial effects, but the mechanism behind the activity of these NPs is not well elucidated yet. However, it is believed that it comprises bacterial cell wall adhesion facilitated by electrostatic interactions. Previously, we have shown the antifungal properties of Cu and its oxide forms [21]. As a follow-up study, in our current work, we investigated some of the mechanisms involved behind the antimicrobial action of Cu NFs. We used commercial as well as Cu NFs synthesized in our laboratory. In this way, we were able to also observe the effect of the nanostructures depending on the concentration and size of the Cu NFs.

Structural organization such as the molecular packing of lipids are intrinsic properties of cell membranes and contribute to biophysical processes, including endocytosis, exocytosis, and autophagy. These are important for cellular function. Introduction of some substances such as NPs may interfere with these vital functions by changing lipid structural organization, thus disrupting normal/expected membrane dynamics. Therefore, studying these structural dynamics provides insight into the physical basis of changes in cellular geometry, which occur during biophysical processes such as membrane invagination. Indeed, different types of stimuli, including physical (heat, light), biomolecules (amyloid beta), and antioxidants (polyphenols) introduced to lipid vesicles, have been shown to induce morphological transformations [22,23]. Giant unilamellar vesicles (GUVs), that is, model membranes with diameters greater than 10 μm, have been used for investigations of the physical and biological properties of vesicle membranes. The cell-sized vesicles allow for the real-time observation of dynamic morphological changes to be clearly visualized without compromising on the “controllable” analytical advantage [24]. They also mimic the physiological environment closely in terms of the spatio-temporal scale at which interactions take place, thus allowing for the understanding and clarification of mechanisms. They have been extensively used in research [25,26]. In our current work, we utilized GUVs to study, in real-time, the spatio-temporal membrane dynamics and changes induced by the presence of Cu NFs. Bhat et al. have studied the role of gold and silver NPs with GUVs [27] in cancer therapies.

Herein, besides demonstrating that our in-house synthesized Cu NFs were antimicrobial and performed better than the commercially soured Cu NFs, we investigated the molecular mechanisms underlying the anti-fungal activities of nanostructured copper and copper oxide. Using biomimetic model membrane systems, we studied in real time the spatio-temporal membrane dynamics induced by the Cu NFs. We observed dynamics with a dominance of large fluctuations, some of which culminated into membrane transformations. Exo-bud/-cytosis was the major membrane change induced by both Cu NFs. Chemical characterization of the functional groups using FTIR revealed that both Cu NFs interfered/interacted with the functional groups in all major biomolecules (lipids, proteins, and carbohydrates). Furthermore, we could visually see the production of a dark pigment. We imagine this to be the production of melanin due to the stress caused by the Cu NFs on the fungus. Melanin production was confirmed and semi-quantified.

## 2. Materials and Methods

### 2.1. Materials

Copper nanoforms (Cu NFs), copper acetate monohydrate, melanin, phosphate buffer saline (PBS) tablets, polyvinyl pyrrolidone (MW 10,000, PVP-10), potato dextrose agar (PDA), and potato dextrose broth (PDB) were obtained from Sigma Aldrich Corporation (Tokyo, Japan) and were used without further purification. Dimethyl sulfoxide (DMSO) was purchased from Wako Pure Chemical Industries, Ltd., Osaka, Japan. Chloroform and methanol were purchased from Kanto-Chemical Co. Inc. (Tokyo, Japan) and Nacalai Tesque Inc. (Kyoto, Japan), respectively. *Colletotrichum gloeosporioides* was a gift from the Plant Pathology Laboratory, Faculty of Agriculture, Kagoshima University, Japan. Membrane lipid 1,2-dioleoyl-*sn*-glycero-3-phosphocholine (DOPC) was purchased from Avanti Polar Lipids, Inc. (Alabaster, AL, USA). All chemicals were of analytical grade and used as received. Deionized water was obtained using a Millipore MilliQ (MilliQ Water, Millipore S.A.S., Molsheim, France) purification system at conductivity 18.2 MΩ cm^−1^. Unless otherwise stated, all analyses were conducted at room temperature (RT).

### 2.2. Synthesis of and Preparation of Copper Nanoforms

Cu NFs were synthesized using a bottom-up approach with copper acetate as a precursor, ascorbic acid as a reducing agent, and PVP-10 as a stabilizer. PVP has been used as a stabilizer in the synthesis of metal NPs including palladium [28]. Briefly, copper acetate (300 mM) and PVP-40 (5% (*v*/*v*)) in MilliQ water were mixed using a magnetic stirrer at ~40 °C for 10 min. Thereafter, 1M of ascorbic acid was slowly added to the solution at 10 μL per min with gentle stirring until the solution changed from bluish to dark red. The dark red precipitate was collected and washed with MilliQ water. This process was repeated twice. Then, the sample was dried in a convection oven at 80 °C for over 12 h. Lastly, the dried sample was ground slightly before analysis. We refer to this sample as the in-house Cu NFs.

### 2.3. Characterization of Synthesized Copper Nanoforms

#### 2.3.1. X-ray Diffraction

Cu NFs were characterized using XRD. The dried sample was fixed on a slide glass for measurement. X-ray diffractograms were obtained using a PANalytical X’PERT PRO MPD diffractometer (Malvern Panalytical, Kobe-shi, Japan) operating at 45.0 kV and 40 mA; the Cu-Kα radiation (λ = 1.54060 Å) was measured at a scan speed of 0.1°/s, step size of 0.01°, and from the 2θ: 20° to 80° range. The commercial Cu NFs were similarly analysed.

#### 2.3.2. Field-Emission Scanning Electron Microscopy (FE-SEM): Energy-Dispersive X-ray Spectroscopy (SEM-EDS)

First, a carbon tape was pasted on the sample holder, and the dried sample was placed on the holder. Excess sample was blown off with a blower, and a coater was used to coat the sample surface with Au. The coating was performed once from directly above the sample and from the oblique direction at 10 mA × 90 s × once using a sputter coater (Polaron SC7610, Fisons Instruments, Saitama-shi, Japan). The synthesized Cu NFs were imaged using FE-SEM (FE-SEM: Hitachi High-Tech Corporation, Model: SU-70, Tokyo, Japan), at an accelerating voltage of 5.0 kV.

Energy-dispersive X-ray spectroscopy (EDS) is a technique used to detect elements in a sample. It is used in conjunction with SEM or TEM. In our study, it was used with SEM. For EDS measurement, pellets (3 mm) were prepared from the dried samples and fixed on a sample holder using carbon tape. Afterwards, the surface of the sample was coated with carbon using a carbon coater (VC-100, Vacuum Device, Ibaraki, Japan). EDS analysis was conducted at an accelerating voltage of 25.0 kV and magnification of 1000× using an Octane Elect Super instrument (AMETEK, Tokyo, Japan).

#### 2.3.3. Dynamic Light Scattering

Immediately after sonication of the Cu NFs, the size distribution and zeta potential of the NPs were analyzed using the DLS technique using a Zetasizer Nano ZS90 (Malvern Panalytical, Japan). All measurements were conducted in water at room temperature (25 °C) and at an approximately 200 kcps count rate.

### 2.4. Preparation of Fungal Culture

A culture of *C. gloeosporioides* was grown on PDA. Fungal isolates were first routinely cultured in PDA in agar plates to build a stock of culture. Agar plates were incubated at 25 °C for 14 d to obtain a good culture. Once the fungi were at an active growth stage, agar blocks were cut and transferred into PDA petri dishes and PDB tubes.

### 2.5. Effect of Copper Nanoforms on Fungal Growth and Structure

The PDA and PDB media were treated with Cu NFs at concentrations ranging from 0 to 1.0 mg/mL. The growth of fungus was measured daily for a period of 14 d using a homemade calibrated ruler as previously reported [24].

For imaging of fungal microstructure, mycelia were harvested after 14 days of incubation (with and without treatment with Cu NFs) from the PDB media and gently collected on filter paper. Thereafter, the mycelia were washed three times with MilliQ water to remove residual PDB. Subsequently, the mycelia were dried at 80 °C for 24 h then ground into fine powder using a mortar and pestle. The sample was fixed on a sample holder using carbon tape. A Quanta 400 instrument (FEI, Thermo Fisher Scientific, Tokyo, Japan) operating in low-vacuum mode was used with water vapor and an accelerating voltage of 15.0 kV.

### 2.6. Chemical Composition of Fungus

To understand how the Cu NFs interacted with the fungus at the chemical level, we used Fourier transform infrared spectroscopy (FTIR) to elucidate the types of bonds involved and any disruptions in the presence of the nanoparticles. FTIR has been used to even discriminate between genus, species, and strain level [29,30].

Mycelia were harvested as in Section 2.5. However, they were subsequently ground after drying and were analyzed using a JASCO FT/IR-4200 instrument (JASCO International Co. Ltd., Tokyo, Japan) with an attenuated total reflection set-up (ATR PRO 410-S). The samples were deposited on the ATR diamond prism (45° angle of incidence), yielding one reflection. Absorption spectra at a resolution of one datapoint for every 1 cm^−1^ were obtained in the region between 4000 and 400 cm^−1^.

### 2.7. Preparation of Cell-Sized Lipid Vesicles

DOPC lipid vesicles were prepared following the natural swelling method [31]. The lipid mixture was dissolved in chloroform:methanol (2:1; (*v*/*v*)) in a Durham glass bottle to a final concentration of 0.2 mM. Glucose in methanol was added to the lipid to give a final concentration of 0.2 mM. The solvent was subsequently removed by evaporating the tube under a gentle nitrogen stream and drying it in a desiccator for 3 h, resulting in a thin lipid film at the bottom of tube. The film was rehydrated with 3 μL of MilliQ water and incubated at 55 °C for 10 min. Afterwards, 197 μL of MilliQ water was added, and the swollen lipid solution was left on a dry heating block overnight at 37 °C for the self-assembly of lipid vesicles.

### 2.8. Observation of Membrane Dynamics

A liposome solution (5 μL) prepared above was placed in silicon well (0.2 mm) on a slide glass and covered with a small cover slip. To study the interaction of NPs with the lipid membrane, 5 μL of the liposome solution and 5 μL of Cu NF prepared in MilliQ water (Cu NPs and Cu_2_O NPs; (1 mg/mL)) were poured into a test tube, gently mixed by soft tapping, and observed soon after. Observation of the vesicular dynamics was within 2 min of the solution’s introduction to the lipid vesicles. As time affects the interaction between the membrane and NPs, we carefully followed the exact same experimental conditions and procedures for each analysis and conducted at least 20 replicates for each type of interaction. Microscope observation was carried out within 3 min of sample mixing. We observed changes in membrane morphology using a phase-contrast microscope (Olympus AX80 and IX70; Olympus, Tokyo, Japan) at RT. The silicon well and cover slip ensured that evaporation of the solution did not occur over the duration of the experiment. Images were recorded on a hard disc.

### 2.9. Detection of Melanin Production

We used a fluorescence-based assay for the detection of melanin [32]. First, the melanin had to be extracted from the fungal culture grown in-PDA incubated (with and without Cu NFs) Petri dishes for a period of 20 days. The fungi, together with the PDA media, were transferred to PBS (pH 7.4) solution and mixed thoroughly. The mixture was heated with constant stirring at approximately 95 ± 5 °C for 20 min and centrifuged at 2000× *g* for 10 min to separate the fungus from the media. The precipitate was then dissolved in 10% (*v*/*v*) DMSO of 1 M NaOH solution using an ultrasonic wave water bath at 75 °C for 4 h. This solution was referred to as “extractable” melanin. After centrifugation at 14,000 rpm for 15 min, 35% (*v*/*v*) H_2_O_2_ solution was added to the supernatant and kept in the dark at RT for 4 h. Fluorescence was measured at excitation 470 nm and emission 550 nm using an Infinite 200 PRO Mplex spectrometer, TECAN, Mannedorf, Switzerland. Standard solutions of melanin were similarly detected, and a standard curve was drawn. Concentrations of extractable melanin were henceforth calculated using the standard curve.

## 3. Results and Discussion

### 3.1. Characterization of Copper Nanoforms

#### 3.1.1. XRD

**Figure 1:**
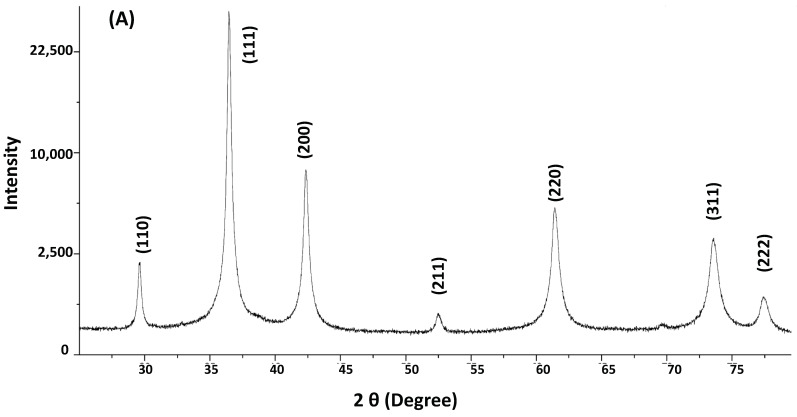

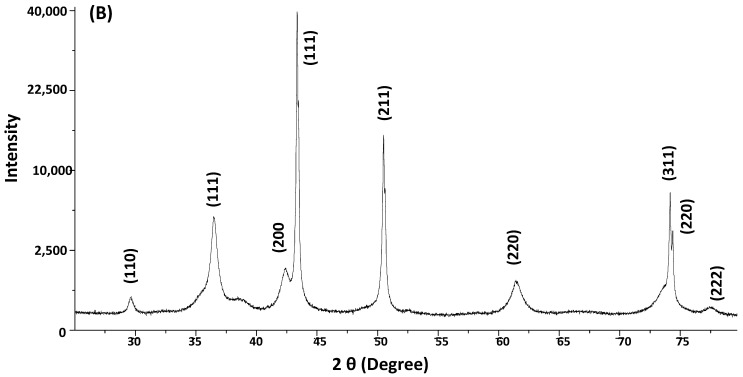
X-ray diffraction (XRD) patterns of (**A**) in-house Cu NFs and (**B**) commercial Cu NFs. Patterns were obtained with a PANalytical X’PERT PRO MPD diffractometer (Malvern Panalytical, Japan) operating at 45 kV and 40 mA with Cu-Kα radiation (λ = 1.54060 Å) measurement being performed at a scan speed of 0.01(units) from the 2θ 20° to 80° range.

The Cu NFs were characterized using XRD spectroscopy. Major sharp diffraction peaks were obtained at 2θ 30°, 36°, 42°, 53°, 63°, 73.5°, and 77° for the in-house synthesized Cu NFs (Figure 1A). These peaks were assignable to lattice planes (110), (111), (200), (211), (220), (310), and (222), respectively, of crystalline Cu_2_O (JCPDS Card No. 04-003-6433). The peaks represent a face-centered cubic structure of Cu_2_O [33]. The diffraction patterns for the commercial Cu NFs were very similar to the in-house ones (Figure 1B) with the exception of an additional not fully resolved diffraction peak at 43°. This corresponds to (111) of cubic symmetry Cu^0^ (JCPDS Card No. 01-070-3039). Another exception is a barely resolved peak (shoulder to peak at 74.1°). This corresponds to (310) of Cu_2_O (JCPDS Card No. 04-003-6433) and the main resolved peak at 74.1° (220) of cubic symmetry Cu^0^ (JCPDS No. 01-070-3039).

Thus, the synthesized Cu NF sample was characterized as a crystalline pure Cu_2_O, and the commercial Cu NF could be identified as a mixture of Cu_2_O and Cu^0^, both of which are crystalline cubic structures.

#### 3.1.2. Energy Dispersive X-ray Spectroscopy (EDS)

**Figure 2:**
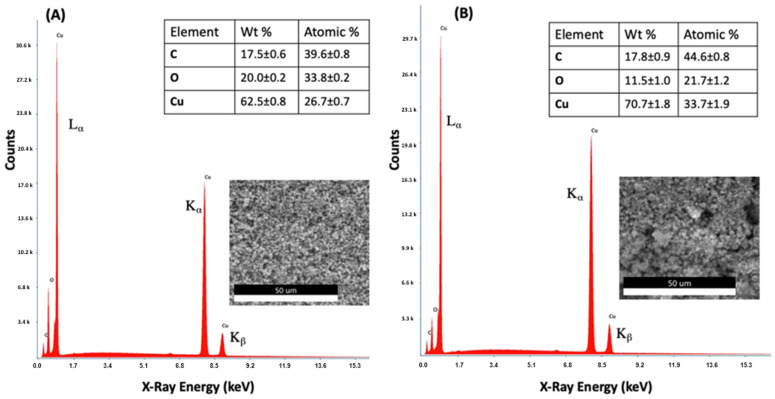
Dispersive energy X-ray spectra of (**A**) Cu NFs synthesized in house using ascorbic acid and polyvinyl pyrrolidone and (**B**) commercial Cu NFs. The elemental (% weight and atomic %), values and SEM images of the analysis area can be seen (inset). The analysis was conducted in triplicate at an accelerating voltage of 25.0 kV and magnification of 1000×. Elemental analysis data are the mean of three replicates.

Energy-dispersive X-ray spectroscopy (EDS) was used in conjunction with SEM to analyze elements in the Cu NFs. EDS measurement is based on the principle that when the elements are subjected to excitation upon being shot by an electron beam, the elements emit/release a specific amount of X-ray energy that is particular to each element. The energy emitted for each element is also related to how far excited electrons in the element fall from upper orbitals to lower ones to fill the vacancy of displaced electrons. The purity of the synthesized Cu NFs is very high indeed, and only three elements were detected. As shown in Figure 2, the spectra of the synthesized and commercial Cu NFs are very similar. They both display typical emission energies for Cu at ~0.93 keV, which corresponds to the fall of electrons from an upper orbital M to L shell (L_α_). The energy spikes at ~8.04 keV and ~8.91 keV correspond to X-ray emissions K_α_ and K_β_, respectively, which were released by electrons as they fell from the L to K electron orbitals. The amount (counts) of released energy is not too different between the two Cu NFs. The commercial Cu NFs had a slightly higher count at 69.7% compared with the in-house Cu NFs (63.3%). In both spectra, carbon was detected. Oxygen element was also detected. We imagine that the O comes from O in the Cu_2_O nanostructures. We imagine that this is contamination from the carbon tape that was used to fix the sample to the sample holder [34]. The micrograph of the synthesized Cu NFs (A) shows that their structures have more evenly distributed surfaces than the commercial nanoforms (B).

#### 3.1.3. Field-Emission Scanning Electron Microscope (FE-SEM)

**Figure 3:**
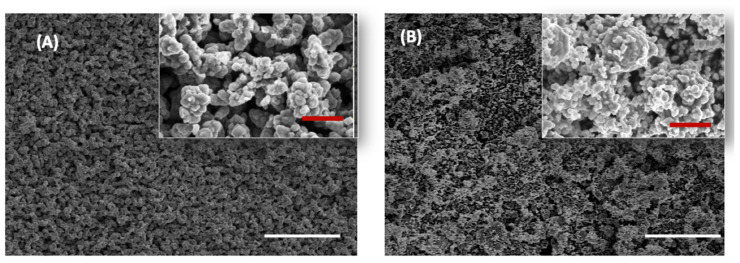
Typical SEM images of Cu NFs (**A**) synthesized in house using ascorbic acid and polyvinylpyrrolidone and (**B**) commercially sourced Cu NFs. Bars indicate 20 μM and 2 μM (insets). The white bars represent 100 μm size; the red bars (inset images) represent 10 μm size Samples were imaged using an accelerating voltage of 5.0 kV.

The structure of Cu NFs was analyzed using field-emission scanning electron microscopy (FE-SEM). The images in Figure 3 show an even size and surface distribution of particles for the synthesized Cu NFs (A) compared with the commercial Cu NFs (B). A closer look at the insets shows smooth, spherically shaped nanoparticles, which are agglomerated into bigger structures (the self-evolution of the agglomerates into grapes-on-stick structures are also interesting in their own right. A study of structural evolution under different conditions is underway and will be reported separately). The nanoparticle agglomeration in both samples is due to the presence of high surface energy. Agglomeration of Cu NFs has been studied. It has been reported that commercially purchased Cu NFs of 40, 60, and 80 nm resulted in agglomerates of 335, 360, and 365 nm in water respectively. The agglomerates were even larger in cell media [35].

### 3.2. Effect of Copper Nanoforms on Fungal Growth and Microstructure of Mycelia

#### 3.2.1. Effect of Copper Nanoforms on Fungal Growth

**Figure 4:**
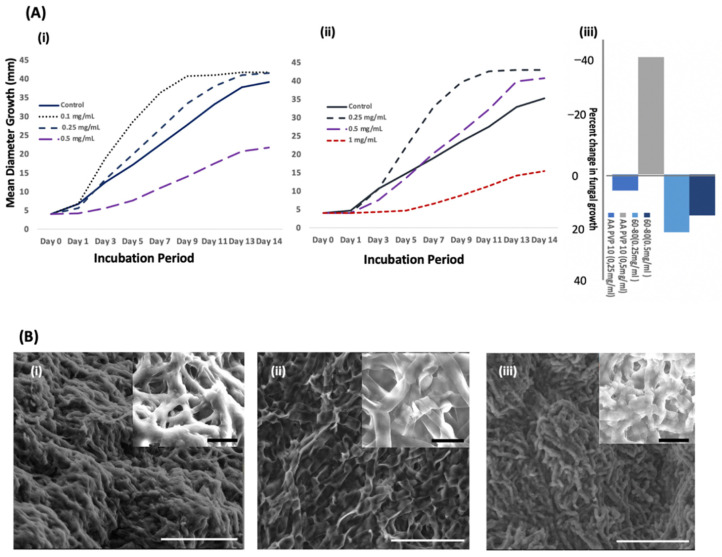
Effect of Cu NFs on *C. gloeosporioides*, a fungus that attacks food crops. (**A**) Diameter growth of the fungus in the presence and absence (control) of (**i**) in-house Cu NFs and (**ii**) commercial Cu NFs; Percent inhibition in fungal growth in the presence of Cu NSFs (**iii**); (**B**) SEM images of the of the fungus (**i**) alone; in the presence of (**ii**) inhouse Cu NFs and (**iii**) commercial Cu NFs. The white bars represent 50 μm size; the white bars (inset images) represent 10 μm size. Images were obtained in low-vacuum mode with water vapor and an accelerating voltage of 15.0 kV.

We exposed *C. gloeosporioides* to the in-house Cu NFs and commercial Cu NFs at different concentrations. As can be seen in Figure 4A, both types had the effect of increasing the growth of the fungus at low concentrations (less than or equal to 0.25 mg/mL). In fact, commercial NFs increased fungal growth even at 0.5 mg/mL at this concentration; we could begin to observe a decrease in growth when the fungus was exposed to in-house NFs. It was only at a higher concentration of commercial NFs (1 mg/mL) that there was a decrease in fungal growth (Figure 4A). Our inhouse Cu NFs were characterized as crystalline Cu_2_O NFs that were center-cubic faced. The commercial NFs were characterized as a combination of crystalline Cu_2_O NFs and Cu NFs. Another difference between the two Cu NFs are their respective sizes. The shape and size of NFs influence how they interact with biological molecules, including cells and organisms (bacteria, fungi, etc.) [36]. At this point, we cannot yet confirm which, if not both, of the parameters (oxidation state and size of NFs) is most effective at inhibiting the growth of the fungus. However, we can note that in our previous work [21], we reported that CuO NPs were more effective at inhibiting the same fungus compared with Cu NFs.

The increase in fungal growth at a low concentration of Cu NFs was very interesting. The same effect has been reported in some bacterial and fungal species in the presence of low concentrations of Cu [37]. Therefore, we decided to examine the overall structure of the mycelium using a simple optical microscope (BA210EINT (Shimadzu, Tokyo, Japan)). Compared with the control, we observed thinner, longer, and less dense mycelia at low concentrations (Figure S1A). These appeared darker in color, and the changes in fungal structure could be seen by the naked eye (Figure S1B). Furthermore, we used a scanning electron microscope to zoom in on the microstructure of the fungus.

#### 3.2.2. Micrographs Fungus after Exposure to Copper Nanoforms

The mycelia of healthy fungus can be seen as a long tubular structure (Figure 4(Bi)). This structure has been flattened, and some tears (broken mycelia) could be clearly observed when the fungus was exposed to in-house Cu NFs (Figure 4(Bii)). Interestingly, treatment of the fungus with commercial Cu NFs resulted in a different macrostructure of the fungus. The mycelia look very short and are somehow clustered in bunches (Figure 4(Biii)). The two forms of Cu NFs were different in their size and chemical structure. Analysis of the in-house Cu NFs by XRD showed that its peaks represented a face-centered cubic structure of Cu_2_O, while the commercial Cu NFs could be identified as a mixture of Cu_2_O and Cu^0^. The SEM images showed both Cu NFs to have agglomerated, and the agglomeration with individual particles were slightly smaller for the commercial sample compared with the in-house sample. Because the EDS data showed both forms to be of similarly good purity with slight contaminations from carbon, we can propose that the differences in the macrostructure of the fungus after treatment with the two Cu NFs could only be due to the size difference and/or the oxidation state of the copper nanostructures.

### 3.3. Interaction of Copper Nanoforms with Fungus: Mechanisms

#### 3.3.1. FTIR Analysis of the Interaction of Copper Nanoforms with Fungus

**Figure 5:**
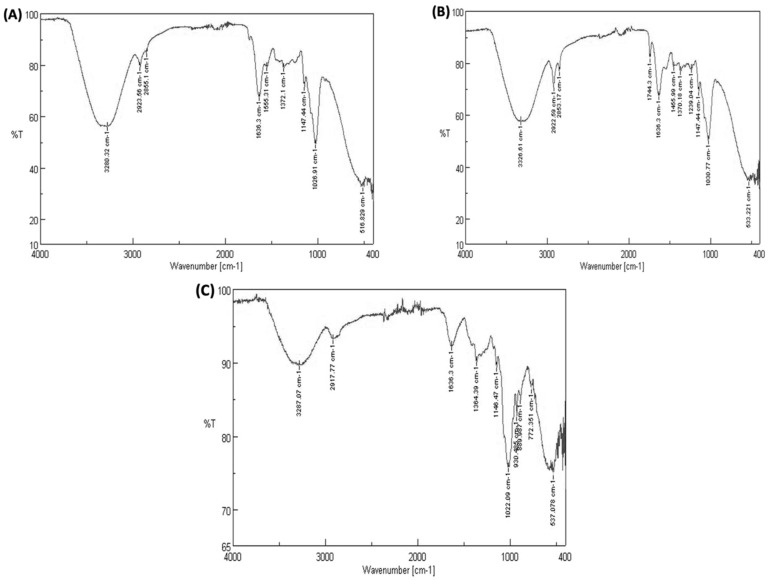
Interaction of *C. gloeosporioides* with copper nanoforms analyzed using FTIR. FTIR spectra of fungal mycelium (**A**) alone (control), (**B**) with Cu NPs, (**C**) with Cu_2_O NPs (FTIR analyses were conducted using a JASCO FT/IR-4200 instrument with an attenuated total reflection (ATR) set-up). Absorption spectra were obtained in the region between 4000 and 400 cm^−1^.

Salman and colleagues provided a thorough characterization of fungal mycelia using FTIR [38]. We have analyzed our results based on that characterization. As can be seen in Figure 5, we observed four major absorbance peaks in all three samples (mycelium only, mycelium treated with Cu NFs, and mycelium treated with Cu_2_O NFs) and minor ones in the fingerprint region. The peak at ~3280 cm^−1^ corresponds to an O–H stretch band from water [38]. Absorbance peaks at 2923 and 2855 cm^−1^ are C–H stretching bands (and a barely visible band at 1748 cm^−1^ due to a C=O stretch) attributed to lipid molecules. At lower wavenumbers within the borders of the diagnostic and fingerprint regions, we can observe absorption bands corresponding to amide (1636 cm^−1^, 1555 cm^−1^) stretches caused by proteins in the sample and chitin. The amidic C–N stretch (1372 cm^−1^) is from chitin and proteins in the sample [39]. In the fingerprint region, we see a small band at 1239 cm^−1^ corresponding to tertiary amine, N–H bending [40], with two bands corresponding to carbohydrate C–O stretches at 1147 cm^−1^ and 1026 cm^−1^ and a barely visible PO_2_ stretch (1075 cm^−1^) occurring due to nucleic acids.

In the presence of Cu NFs, the FTIR spectra appear to be very similar to the control with only a small peak, possibly due to methylene scissoring vibrations from the proteins in the solution, and the peak appears at a 1455 cm^−1^ wavenumber [41]. Cu NPs have been shown to be antibacterial due to their affinity for highly abundant amines and C=O on the cellular surface [42]. Thus, another possibility is that the strong interaction of Cu NPs and amide may have caused a band displacement. This minor vibration was not automatically detected in the control sample but can be observed by the eye similarly to the Cu NFs-treated sample. Peaks at 1744 cm^−1^ and 1239 cm^−1^ are more prominent here than that in the control. It indicates a decrease in the concentration of C=O of lipids showing affinity towards Cu NPs.

Of further interest is the total absence of an absorbance band at 1555 cm^−1^ due to amides from proteins [39,41] and chitin [39]. In addition, there are three more bands in the fingerprint region at 930 cm^−1^, 889 cm^−1^ (P–O stretching from polyphosphates in the cell wall), and 772 cm^−1^. Here, an increase in the number of bands shows the masking of all of the biomolecules by Cu_2_O NFs, which indicates a decrease in the concentration of the biomolecules after interaction with Cu_2_O NFs.

Using a different fungal strain, we could still observe the same four major absorption bands in fungal mycelium only and in mycelium treated with Cu NFs, as well as minor ones in the fingerprint region (Figure S2 in Supplementary Materials). The peak at ~3320 cm^−1^ corresponds to an O–H stretch band from water [38]. Absorbance peaks at 3008 and 2922 cm^−1^ and 2853 cm^−1^ are C–H stretching bands from Csp2-H, Csp3-H, and Csp3-H, respectively; a barely visible band at 1745 cm^−1^ is due to C=O stretch attributed to lipid molecules. In the fingerprint region, we see two bands corresponding to carbohydrate C-O stretches at 1148 cm^−1^ and 1026 cm^−1^. In the presence of Cu NFs, the FTIR spectra appears to be very similar to the control.

#### 3.3.2. Aggregation and Localization of Copper Nanoforms in Lipid Vesicles

**Figure 6:**
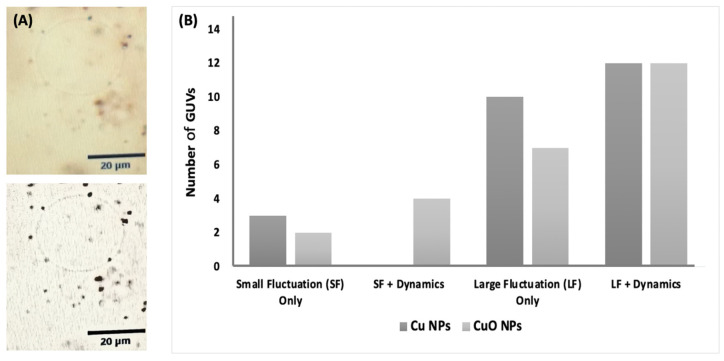
(**A**) Aggregated Cu NFs in aqueous lipid media (black dots). The outline of a lipid vesicle can be seen. Giant unilamellar vesicles were prepared using the 1,2-dioleoyl-*sn*-glycero-3-phosphocholine (DOPC, 0.2 mM) by the natural swelling method. Top: original image; Bottom: image after artistic modification to enhance contrast; (**B**) membrane dynamics induced by 0.5 mg/mL of Cu NPs and Cu_2_O NPs. Vesicles were prepared as in (**A**).

First, we introduced 1.0 mg/mL of Cu NFs and Cu_2_O NFs into separate DOPC GUVs to provide final metal and lipid concentrations of 0.5 mg/mL and 0.2 mM, respectively. Upon observation under phase contrast microscopy, we could clearly observe Cu NFs with sizes well above what was supplied (quoted by supplier as 60 nm) (Figure 6A). This shows that the Cu NFs were not stable and had aggregated in the biological environment. These observations are in agreement with previous reports and that the NPs aggregated in the aqueous environment, as the zeta potential of both Cu NFs and Cu_2_O NFs samples were very low at 0.05 mV and −0.05 mV [41]. Furthermore, the size distribution of the “aggregated” Cu and CuO NFs averaged 175.6 nm and 254.8 nm, respectively, with a wide spread between 80 and 850 nm (Figure S3). In this experiment with lipid vesicles, some of the observed aggregated Cu NFs were larger than 1 μm. Indeed, agglomeration of Cu NFs in cells and cell media have been reported to be higher than in water [35].

Both forms of Cu were observed outside of the lipid vesicles. In real time, the aggregated nanoforms were captured bouncing on and off of lipid vesicles. We imagine that there was a repulsion between the hydrophilic head groups of the lipid molecules and the aggregated copper nanoforms through sheer steric repulsion. As there is only a slight negative charge on the phosphate group, a very slight negative charge (−0.05 mV) on the surface of the Cu_2_O NFs, and no charge on the Cu NFs, it seems unlikely that the effect was due to charge. Hydrophobicity of the copper nanoforms, especially in aggregated forms, may also have hindered a direct interaction with the lipid headgroups (the nanoparticles were prepared as a suspension in MilliQ water. That is, they did not dissolve). What we were able to observe using a simple optical microscope was mainly relatively large particles. A focus on the lipid vesicles themselves showed that the vesicles were destabilized in the presence of the nanoparticles. This has been observed with amyloid beta fibrils upon interaction with lipid vesicles. The fibrils were observed to not closely associate with lipid vesicles. Localization of labelled oligomeric amyloid beta species could be seen on the vesicular surface [23]. This could be due to the discussed possible stearic repulsion between the vesicles and the vesicles and/or direct interaction between the vesicles and smaller (<100 nm) nanoparticles not visible under the microscope used.

#### 3.3.3. Spatio-Temporal Membrane Dynamics Induced by Copper Nanoforms

There are two oppositely acting major forces existing among the phospholipids in the interfacial region of the lipid bilayer that control the surface area (*A*) per molecule. There is a negative pressure (also known as interfacial tension) from the hydrophilic–hydrophobic interface as a result of the hydrophobic effect; the positive pressure is due to repulsion between the phospholipid headgroups. The former force, which is generated from interfacial tension among identical molecules, causes the molecules to assemble and decreases the surface area *A*. In contrast, the latter, which includes steric repulsive interaction, hydration force, and electrostatic double-layer contribution, tends to disaggregate the molecules and increases *A* [43]. Changes in the balance between the two forces significantly influence membrane area and dynamics.

We investigated the effect of Cu NFs and Cu_2_O NFs upon interaction with DOPC lipid vesicles by observing, in real time, the membrane instability characterized by fluctuation (undulation) and/or membrane transformation. Both Cu and Cu_2_O nanoforms induced membrane fluctuation for all vesicles (*n =* 25). This suggests that there was an increase in the surface area *A* to inner volume *V* ratio [23]. The fluctuations were initially small. Eighty-eight percent (*n =* 22) of total lipid vesicles increased the intensity of the undulations in the presence of both copper forms (Figure 6B). Although only marginally different, Cu_2_O NFs seemed to have had a higher effect on membrane dynamics than the Cu NFs. They also agree with the effect on fungal growth and observed images of *C. gloeosporioides* after exposure to the coppers (Figure 5).

**Figure 7:**
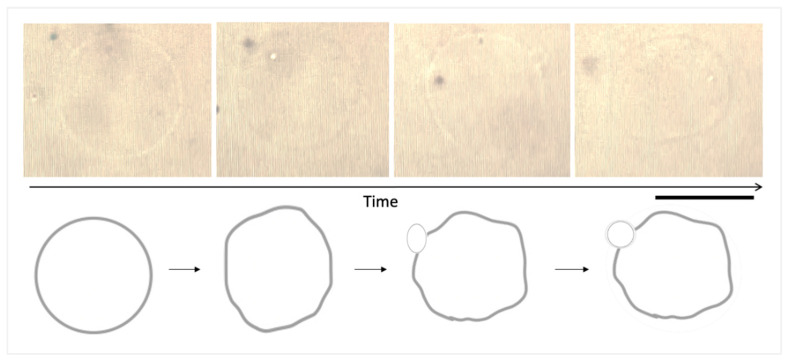
Endo-bud formation. The most dominant lipid vesicular transformation pathway induced by Cu NFs. Top: Original image; Bottom: stylized image to clearly show what was observable under the objective lens. Giant unilamellar vesicles were prepared using 1,2-dioleoyl-*sn*-glycero-3-phosphocholine (DOPC, 0.2 mM) by the natural swelling method. Size bar = 20 μm.

Some of the lipid vesicles exhibited membrane transformations, forming exo-buds via large membrane fluctuations as the most dominant pathway (Figure 7). Exocytosis and product endo-bud formation by membranes are normal biological processes, such as the release of large molecules from within the cell into the extracellular space. It can also be a way of eliminating of unwanted molecules adsorbed or attached to the membrane surface. It is a form of transport. In this study, the majority of the membrane dynamics were exo-bud/-cytosis. In biophysical terms, we imagine two possible scenarios: (i) adsorption of small-sized Cu NFs and Cu_2_O NFs to the membrane surface; and/or (ii) membrane penetration of even smaller-sized NPs. Upon these events, the lipid bilayer is destabilized as observed by the membrane fluctuations and ensuing dynamics. These membrane dynamics are a result of the increase in the surface area to volume ratio. The membrane tries to regain equilibrium by reducing its effective surface area. Indeed, the opposite dynamics (endo-bud/-cytosis) would also similarly reduce the effective surface area and enable equilibrium to be re-attained. In biological terms, we begin to imagine how the presence of Cu and Cu_2_O NFs, especially the very small (non-aggregated forms or small aggregates) forms, can cause membrane exocytosis as a means to eliminate any adsorbed and penetrated materials on the cell surface. Exocytosis of NPs has been reported [44]. Although not a major dynamic, endo-bud/cytosis was also observed. Similarly, we can imagine that this would most likely be a means by which small nanoparticles would gain entry into the cell through clathrin-mediated endocytosis. Our future research will focus on (i) measuring the size distribution and zeta potential of the copper and copper nanoforms in aqueous solutions containing lipid vesicles; (ii) studying their localization in lipid vesicle solutions and using higher-resolution microscopy; and (iii) labelling the vesicles to investigate any phase changes and quantify membrane fluidity. Furthermore, the observed *hit-and-bounce-off* effect of the aggregated copper forms on the membrane surface could be studied deeply to understand their immediate effect on membrane curvature.

#### 3.3.4. Production of Melanin by Fungus

**Figure 8:**
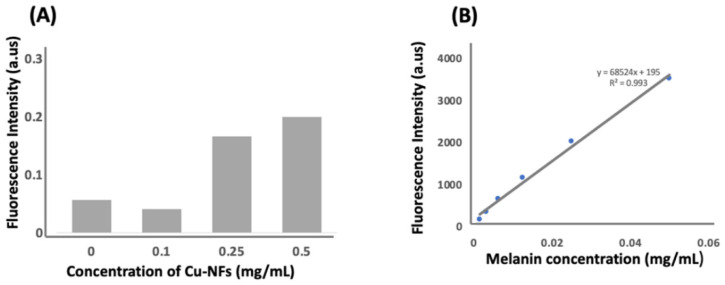
Melanization of *C. gloeosporioides* in the presence of in-house Cu NFs (**A**). *C. gloeosporioides* was cultured in potato dextrose agar for 14 d at 25 °C in the presence of in-house Cu NFs. Standard curve of melanin (**B**).

In this section, we discuss the fungal response upon exposure to the Cu NFs. This was very interesting for us as we did not set up our experiment to investigate melanin production. During the experiments on the effect of Cu NFs on growth of *C. gloeosporioides,* we observed pigment formation. We believed it to be melanin, as melanin has been reported to be produced by organisms including fungus [45] in response to stress. The exposure to Cu NFs must have induced a defense mechanism in the fungus. The degree of melanization was determined using a hydrogen peroxide-based assay [32]. The results show that melanin was produced in a concentration-dependent manner, that is, the higher the concentration of Cu NFs, the bigger the amount of melanin produced (Figure 8A). An estimate of the melanin produced was calculated from a standard curve of melanin (Figure 8B). In the absence of Cu NFs, the fungus produced 0.057 mg/g of melanin. This amount changed to 0.041 mg/g, 0.166 mg/g, and 0.199 mg/g in the presence of 0.1 mg/mL, 0.25 mg/mL, and 0.5 mg/mL of Cu NFs.

At a low concentration of Cu NFs, melanization was lower than the control. This is a similar trend to the effect on fungal growth. We can imagine that the low concentration of Cu NFs is in fact a stimulant to fungal growth rather than a stress factor. This observation is in agreement with a report on how some species of bacteria and fungus perform better in the presence of low levels of copper [37].

## 4. General Summary

In this research, we investigated the interaction of copper nanoforms (Cu NFs) with a plant-devastating pathogen *C. gloeosporioides.* The details and discussion of the findings are in the previous section. To summarize, we synthesized Cu NFs from copper acetate using a top-down approach, with ascorbic acid acting as a reducing agent and polyvinylpyrrolidone 10,000 (PVP-10) acting as the stabilizing agent. We also purchased Cu NFs. We characterized both Cu NFs using EDS, and the results of the in-house and commercial Cu NFs were very similar in terms of purity (Figure 2). Both were of very high purity. Only carbon was noted as a contaminant in both samples, most likely originating from the carbon tape used to fix the sample onto the sample holder. Similar sources of contamination have been reported [34]. This shows that the protocol that we used to synthesis our Cu NFs was very robust. Furthermore, the in-house Cu NFs showed a better even distribution of structures compared with the commercial Cu NFs. In both nanomaterials, there was a degree of agglomeration. The agglomerates, as well as individual particles, were smaller for commercial Cu NFs, as can be seen in the SEM images (Figure 3). XRD was used to understand their chemical properties. Both Cu NFs were characterized as having a cubic crystalline structure. However, the in-house Cu NFs were a cubic structure of Cu_2_O, while the commercial Cu NFs could be identified as a mixture of Cu_2_O and Cu^0^. In short, we could synthesize high-purity Cu NFs using ascorbic acid as a reductant and PVP-10 as a stabilizer.

We studied the antimicrobial activity of the Cu NFs on *C. gloeosporioides*. The growth of the fungus in the presence and absence of the NFs was measured daily for 2 weeks. In addition, simple optical microscopic and SEM images of the fungus were obtained at the end of the incubation period. The following are some of the notable findings: (i) The in-house Cu NFs were more effective antimicrobial agents than commercial NFs. The difference could be attributed to the differences in size and/or oxidation states of the Cu NFs; (ii) At low concentrations of Cu NFs (0.25 mg/mL for in-house and up to 0.5 mg/mL for commercial NFs, the fungal growth was faster, but the mycelial density was less than the control. In the near future, we will investigate why the low concentration of Cu NFs boost the growth of fungus, albeit seemingly having a weaker vegetative form; (iii) Melanization of the fungus, in response to the presence of Cu NFs was observed and quantified. It corresponded to the concentration of the added Cu NFs.

Lastly, we studied some of the possible mechanisms behind the antimicrobial activity of Cu NFs. The localization of aggregated Cu NFs could be clearly observed outside the model membranes. Spatio-temporal membrane dynamics (fluctuation and membrane transformation) were captured in real-time. The dominant dynamics culminated into large fluctuations. Some of the large fluctuations resulted in vesicular transformation. The major transformation was exo-bud/exo-cytosis.

## Data Availability

Data are contained within the article and Supplementary Materials.

## Supplementary Materials

The following supporting information can be downloaded at: https://www.mdpi.com/article/10.3390/nano13232990/s1, Figure S1: Effect of Cu-NFs on *C. gloeosporioides*, a fungal pathogen that attacks food crops (A) image of fungus (i) alone (Control); and in the presence of (ii) in-house Cu-NFs, (iii) commercial Cu-NFs, taken using an optical microscope at ×25 magnification; (B) Photos of the fungus (i) Control; and in the presence of (ii) inhouse Cu-NFs (0.5 mg/mL) and (iii) commercial Cu-NFs (0.5 mg/mL); Figure S2: Interaction of *C. gloeosporioides* with Cu-NFs analysed using FTIR Spectra of fungal mycelium. FTIR analyses were conducted as described earlier; Figure S3: Size distribution of (a) aggregated copper and (b) copper oxide nanoforms analysed by DLS technique using a Zetasizer Nano ZS90 (Malvern Panalytical, Japan). All measurements were conducted in water at room temperature (25 °C) and t approximately 200 kcps count rate.
