# Supplementary material for: Antifungal Activity and Molecular Mechanisms of Copper Nanoforms against Colletotrichum gloeosporioides
Source: Nanomaterials (Basel). 2023 Nov 22;13(23):2990. doi: 10.3390/nano13232990 (PMC10708171; doi:10.3390/nano13232990)
Supplement: Supplementary file 1 [file nanomaterials-13-02990-s001.zip › nanomaterials-2698015-supplementary.pdf]

Antifungal Activity and Molecular Mechanisms of Copper Nanoforms against *Colletotrichum gloeosporioides*

Mun'delanji C. Vestergaard [1,*], Yuki Nishida [1], Lihn T.T. Tran [1], Neha Sharma [2], Xiaoxiao Zhang [1], Masayuki Nakamura [1], Auriane F. Oussou-Azo [3] and Tomoki Nakama [1]

1 Faculty of Agriculture, Kagoshima University, Kagoshima City 890-0065, Japan; k8015687@kadai.jp (Y.N.); tranthaolinh030797@gmail.com (L.T.T.); xiaoxiao8812@icloud.com (X.Z.); masa@agri.kagoshima-u.ac.jp (M.N.); k4618101@kadai.jp (T.N.)
2 Faculty of Fiber Science and Engineering, Kyoto Institute of Technology, Matsugasaki, Sakyo,
Kyoto 606-8585, Japan; neha-sha@kit.ac.jp
3 United Graduate School of Agricultural Sciences, Kagoshima University, Kagoshima City 890-0065, Japan; oauriane1@gmail.com
* Correspondence: munde@agri.kagoshima-u.ac.jp

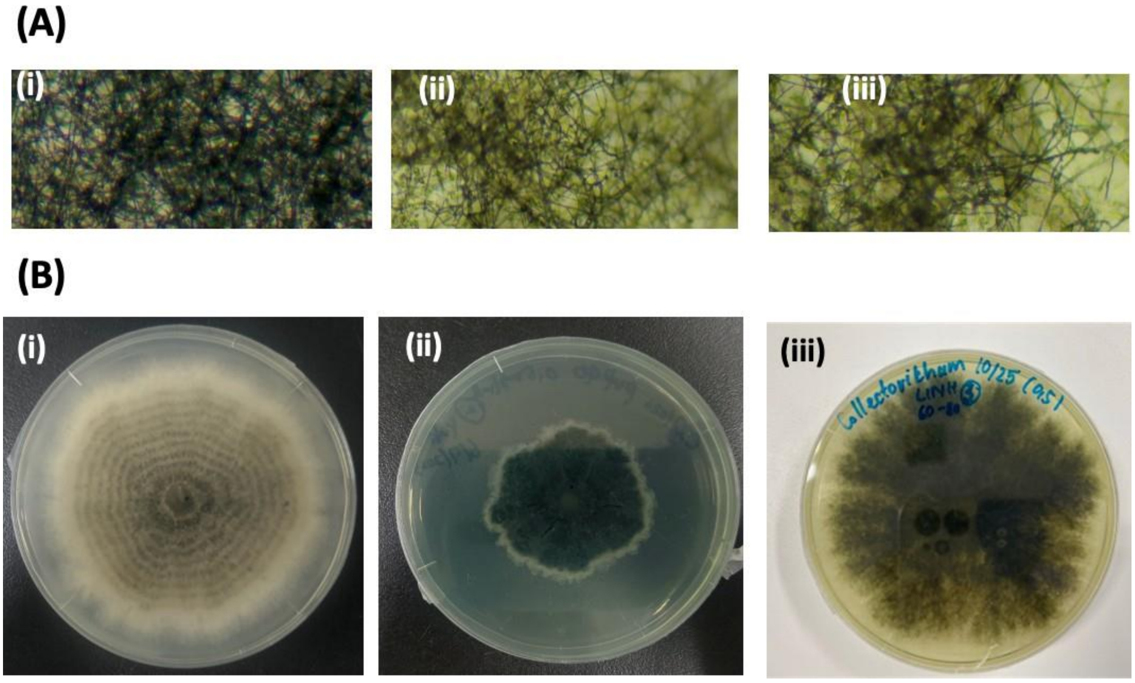

Figure S1. Effect of Cu-NFs on *C. gloeosporioides*, a fungal pathogen that attacks food crops **(A**) image of fungus (i) alone (Control); and in the presence of (ii) in-house Cu-NFs, (iii) commercial Cu-NFs, taken using an optical microscope at x25 magnification; **(B)** Photos of the fungus (i) Control; and in the presence of (ii) inhouse Cu-NFs (0.5mg/mL) and (iii) commercial Cu-NFs (0.5 mg/mL)

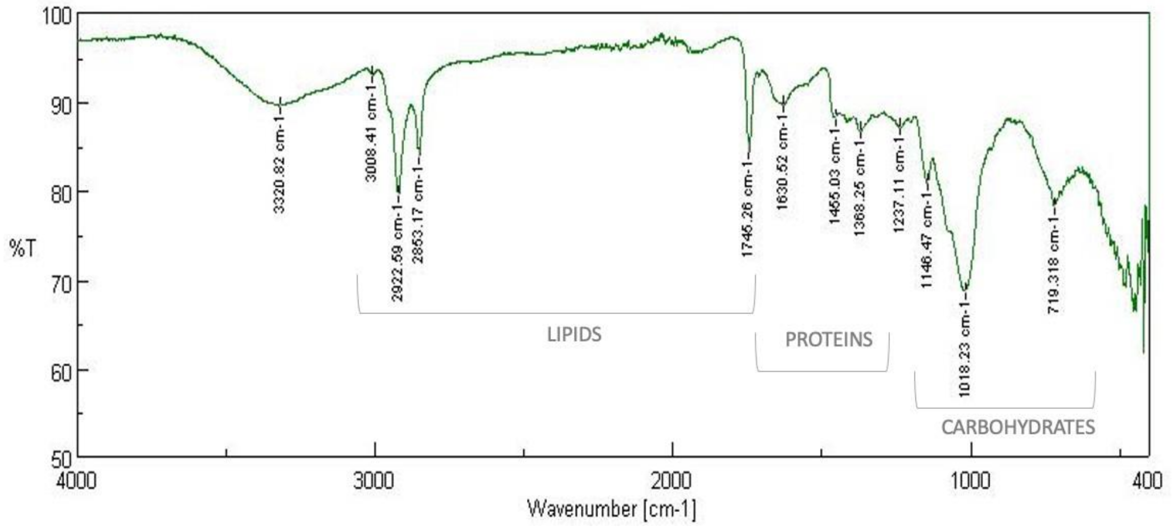

Figure S2. Interaction of *C. gloeosporioides* with Cu-NFs analysed using FTIR Spectra of fungal mycelium. FTIR analyses were conducted as described earlier.

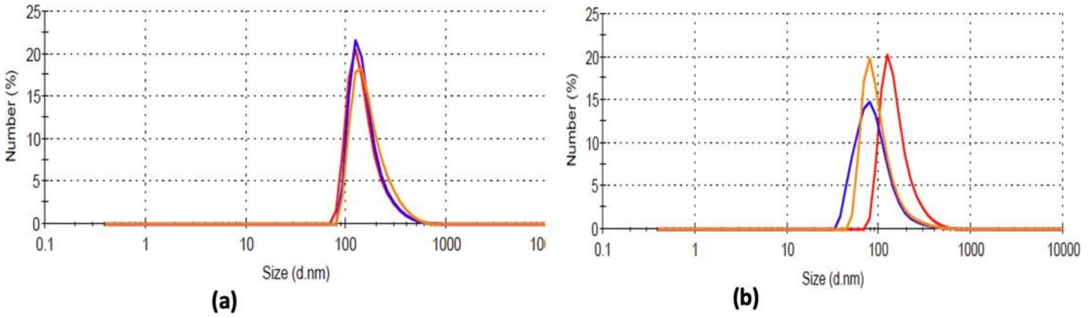

Figure S3. Size distribution of (**a**) aggregated copper and (**b)** copper oxide nanoforms analysed by DLS technique using a Zetasizer Nano ZS90 (Malvern Panalytical, Japan). All measurements were conducted in water at room temperature (25 ^{o}C) and t approximately 200 kcps count rate.
